# Positive pressure ventilation improves oxygen saturation at altitude during recreational aviation: A pilot study

**DOI:** 10.14814/phy2.70210

**Published:** 2025-02-04

**Authors:** Jenna L. Taylor, Aidan K. Downs, Crystal L. Danner, J. Hunter Downs, Josh Donkor, Jessica I. Johnston, Douglas Rozendaal, Peter L. Larsen, Bruce D. Johnson, Douglas T. Summerfield

**Affiliations:** ^1^ Human Integrative and Environmental Physiology Laboratory, Department of Cardiovascular Medicine Mayo Clinic Rochester Minnesota USA; ^2^ Physiology and Ultrasound Laboratory for Science and Exercise (PULSE) School of Human Movement and Nutrition Sciences, The University of Queensland Brisbane Queensland Australia; ^3^ MercyOne North Iowa Medical Center Mason City Iowa USA; ^4^ PlaneLease LCC Mason City Iowa USA

**Keywords:** cerebral blood flow, reaction time, respiratory rate, tidal volume

## Abstract

Aim

We investigated whether a commercial bi‐level positive airway pressure (BPAP) device, would improve peripheral oxygen saturation (SpO_2_) during recreational aviation up to 12,500 feet without supplemental oxygen. Ten adults with recreational flight experience (age:47 ± 14; female = 5) completed a standardized flight profile in an unpressurised aircraft, involving randomized crossover design at 8,000 feet and 12,500 feet with BPAP or control. SpO_2_, middle cerebral artery velocity (MCAv), heart rate (HR), respiratory rate (RR), and relative tidal volume (TV) index were measured continuously. Psychomotor vigilance test (3‐min) assessed reaction time halfway through taxi and altitude stages. Altitude significantly (*p* < 0.05) decreased mean SpO_2_, MCAv, and RR, and increased TV index and HR. There was no effect of altitude (*p* > 0.05) on reaction time. BPAP increased mean SpO_2_ at 8,000 feet [Control: 92 ± 1%; BPAP: 94 ± 2%; mean difference (MD) = 2 ± 2%; *p* = 0.002] and 12,500 feet [Control: 86 ± 4%; BPAP: 89 ± 4%; MD = 2 ± 3%; *p* = 0.013]. BPAP lowered MCAv at 8,000 feet [Control: 53 ± 10 cm/s; BPAP: 50 ± 9 cm/s; MD = ‐3 ± 2 cm/s; *p* = 0.001] and 12,500 feet [Control: 52 ± 10 cm/s; BPAP: 50 ± 8 cm/s; MD = ‐2 ± 3 cm/s; *p* = 0.041]. BPAP increased TV index at 8,000 feet (Control: 6.6 ± 1.3; BPAP:8.1 ± 1.8; MD = 1.9 ± 0.8; *p* < 0.001) but not 12,500 feet, without effect on RR or reaction time. This study provides preliminary results that BPAP may improve mean SpO_2_ for recreational aviators up to 12,500 feet without supplemental oxygen.

## 1 INTRODUCTION

Current United States Federal Aviation Administration (FAA) guidelines for general recreational aviation do not require supplemental oxygen for flight crew at cabin altitudes of 12,500 feet (3810 m) or below (Federal Aviation Administration, 2023). Although the FAA encourages the use of oxygen above 10,000 feet during the day and above 5000 feet (1524 m) at night (Federal Aviation Administration, 2022), pilots may choose not to use supplemental oxygen due to cost and inconvenience. This choice may potentially lead to the dangerous situation of general aviation pilots operating aircraft in a hypoxic state. The FAA defines hypoxia as “a state of oxygen deficiency in the body sufficient to impair function of the brain and other organs” (Federal Aviation Administration, 2022). This can result from inadequate oxygen delivery to the tissues either from low oxygen pressure of the blood (hypoxaemia) or impaired blood supply (Leach & Treacher, 1998). The effects of hypoxia are acknowledged as early as 384 B.C.E. by Aristotle (West, 1998). Signs and symptoms of hypoxia in the average healthy individual typically set in around 10,000 feet (3048 m) and include (but are not limited to) hyperventilation, diaphoresis, nausea, headache, light‐headedness, inability to think clearly, impaired motor control, drowsiness, irritability, and/or euphoria (Harding & Mills, 1983; Pilmanis & Sears, 2003; West, 1998). Some individuals may experience these effects at higher or lower altitudes depending on susceptibility differences (Harding & Mills, 1983). Previous investigations into aviation hypoxia have revealed an increase in pilot procedural errors, as well as deficits in reaction time and ability to perform psychomotor tasks at altitudes of 8,000 feet (2438 m) and above (Denison et al., 1966; Kelman & Crow, 1969; Nesthus et al., 1997).

Alveolar partial pressure of oxygen (PAO_2_) and its relationship to barometric pressure is described by the Alveolar Gas Equation (Petersson & Glenny, 2014; Wagner, 2015). With increases in altitude, PAO_2_ decreases due to the decrease in atmospheric pressure, which in turn reduces arterial PAO_2_ and can cause hypoxaemia (Petersson & Glenny, 2014). Increasing the barometric pressure of inspired air entering the lung system can therefore increase PAO_2_. Moreover, an increase in ventilation can help to raise or maintain PAO_2_ and PaO_2_ (Wagner, 2015). The concept of pressure breathing to maintain PAO_2_ (altitude protection) has been used by fighter jet pilots since the end of World War II (Lauritzsen & Pfitzner, 2003; Pilmanis & Sears, 2003).

In the medical setting, the goal of noninvasive positive pressure ventilation is to maintain optimal levels of PaO_2_ and arterial partial pressure of carbon dioxide (PaCO_2_) while reducing or eliminating the work of the inspiratory muscles (British Thoracic Society, 2002). Continuous positive airway pressure (CPAP) delivers a constant pressure of air into the lungs, which during expiration provides positive end‐expiratory pressure, increasing the pressure within the alveoli above that of atmospheric pressure (Duncan et al., 1986). Bi‐level positive airway pressure (BPAP), combines the benefit of CPAP during expiration with spontaneous assisted breathing or pressure support during inspiration to further increase tidal volume (TV) and the enhance distribution of air within the lungs as well as improving ventilation and facilitating CO_2_ removal (Alviar Carlos et al., 2018). By increasing TV and the barometric pressure of ambient air with BPAP, a pilot's oxygen saturations should increase without the use of supplemental oxygen. Demonstrating this definitively could be beneficial to aviators as the use of noninvasive positive pressure ventilation to increase PAO_2_ may be enough to increase their oxygen saturations and reduce the risk of negative effects of altitude, without the need for cumbersome oxygen cylinders. The potential convenience of a BPAP machine over supplemental oxygen is that it could remain within the plane (e.g., no re‐filling), would require less storage space, less ongoing costs once the device is purchased, and less explosive risk.

We hypothesize that the added pressure from BPAP at altitude would increase the barometric pressure of ambient air into the lungs, thus increasing alveolar oxygen levels and peripheral oxygen saturation without the use of supplemental oxygen. We expect this increased pressure from BPAP would also increase TV (Alviar Carlos et al., 2018). However, previous work in animals (Beecher et al., 1943; Braunwald et al., 1957; Carr & Essex, 1946; Maloney Jr. & Whittenberger, 1957; Qvist et al., 1975) and humans (Hobelmann Jr et al., 1975) has found positive pressure breathing can cause reductions in arterial pressure and cardiac output due to increased pulmonary pressures and reduced venous return to the heart. Although these studies were not conducted at altitude, this indicates we may find concomitant reductions in haemodynamics with BPAP such as cerebral blood flow. Previous work has also shown that flying at altitude without supplemental oxygen may contribute to impairments in cognition (e.g., procedural errors) (Nesthus et al., 1997). Therefore, it is also of interest whether reductions in oxygen saturation at altitude contribute to impairments in cognition such as reaction time, and whether improving oxygen saturation with BPAP may mitigate this.

The primary purpose of this study was to investigate whether reductions in peripheral blood oxygen saturation (SpO_2_) with altitude in an unpressurized aircraft can be mitigated with the use of noninvasive positive pressure ventilation in the form of BPAP (a commercially available device used to treat sleep apnoea) without the use of supplemental oxygen. We hypothesized that the use of BPAP would increase SpO_2_ at altitudes regularly experienced by general aviators. Secondary outcomes will assess the reduction in SpO_2_ and other physiological effects (e.g., cerebral blood flow and reaction time) at general aviation altitudes up to 12,500 feet (3810 m) in an unpressurised aircraft without supplemental oxygen, which at present is allowed by the FAA.

## 2 METHODS

### 2.1 Participants

This study was approved by the Institutional Review Boards from Mayo Clinic and MercyOne North Iowa Medical Center. Male and female adults planning to undertake a flight in an unpressurized aircraft were recruited through the Mason City General Aviation Enthusiast Community in Iowa, United States. Inclusion criteria included adults aged 18–89 years with recreational flight experience above 8,000 feet (as a pilot or passenger). Participants were excluded if they were pregnant, had previously experienced any serious adverse effects during general aviation (e.g., vertigo, loss of consciousness, illness requiring medical treatment), or had any known history of cardiac disease, pulmonary disease, cerebrovascular disease, neurological disease, vertigo, or syncope. All participants provided written informed consent prior to study participation.

### 2.2 Study design

**FIGURE 1:**
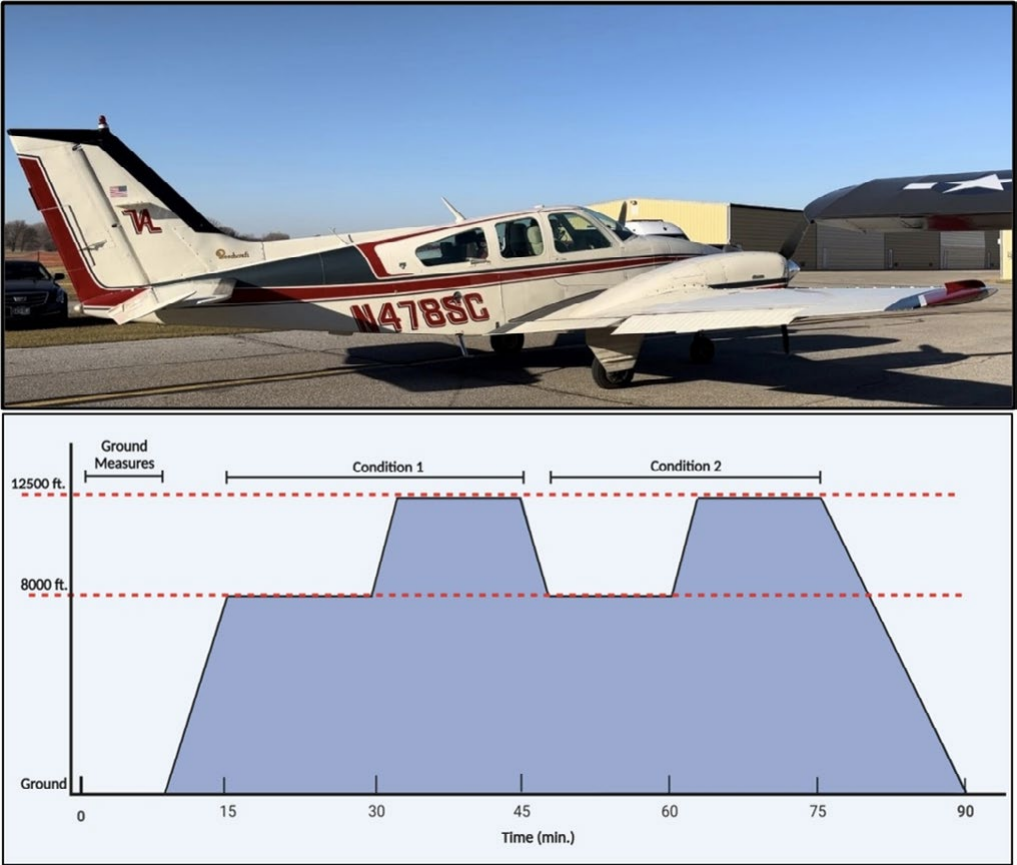
Aircraft (1974 Beachcraft Baron 55) and Flight Profile. Top: Aircraft; Bottom: Flight profile.

The study employed a randomized cross‐over study design, to compare BPAP and control at 8,000 feet and 12,500 feet. Participants completed a standardized flight profile as passengers in an unpressurized aircraft (1974 Beachcraft Baron 55) involving 2 × 15 min periods at 8,000 feet (2438 m)and 12,500 feet (3810 m) (Figure 1). Participants were randomized to either BPAP or control (Condition 1) during the first half of the flight profile, and then performed the other (Condition 2) during the second half of the flight profile. The randomization sequence was generated using a website‐based application (www.sealedenvelope.com). There were two participants per flight and five flights planned over 2 days with optimal weather conditions. During the BPAP condition, the BPAP device (Respironics, Phillips, The Netherlands) was set to 10 cmH_2_0 inspiratory positive airway pressure (IPAP) and 5 cmH_2_0 expiratory positive airway pressure (EPAP).

Participants were instrumented with equipment in the aircraft hangar at a ground level of 1214 feet (370 m). Prior to the flight, participants completed basic spirometry testing (CPFS/D USB Spirometer, MGC Diagnostics, MN, USA) and body fat percentage was measured using bioelectrical impedance analysis scales (Model BF‐681, Tanita, Tokyo, Japan). Participants also became familiarized with the cognitive tests. The following physiological measurements were recorded continuously throughout the flight. Heart rate (HR) and SpO_2_ were measured using a portable pulse oximeter (WristOx 2, Model 3150, Nonin Medical, MN, USA). Middle cerebral artery velocity (MCAv) was measured using transcranial doppler ultrasound with a 2 MHz probe placed over the temporal ultrasound window and secured in place with an adjustable headset (ROBOTOC9MD lunchbox, Multigon Industries, NY, USA). An optimal MCAv signal was obtained using standardized guidelines for depth, velocity, and waveform characteristics (Willie et al., 2011), and settings remained constant during the entire study. Respiration rate and relative TV index (using changes in thoracic and abdominal circumferences) were measured using dual band respiratory inductance plethysmography (Model TN1132/ST, Respiratory Belt Transducer Series, ADInstruments, CO, USA). A data acquisition device and software (PowerLab and LabChart, ADInstruments, CO, USA) was used for recording of the MCAv waveforms and respiratory inductance plethysmography signals. Participants completed a 3‐min psychomotor vigilance test, to measure reaction time, which has previously been shown to be sensitive to deficits from sleep loss and alcohol in aviation research (Benderoth et al., 2021). To administer the test, participants used an iOS iPad application (PVT Research Tool, Texas A&M University System CSE, 2019) on separate devices during flight taxi and halfway through each 15‐min altitude period. This test involved tapping a red dot on the screen as quickly as possible once it appeared and returning the hand to the side of the iPad between attempts.

### 2.3 Data analysis

Data were exported from LabChart and the pulse oximeter device for off‐line analysis. Data for SpO_2_ and HR were time‐stamped for comparison with the flight tracker time and altitude data (FlightAware, website: www.flightaware.com). LabChart comments were used to denote the start and end times for each data analysis period. Mean MCAv was calculated in LabChart using a weighted mean of the waveform envelope. Data were visually inspected for artifact, with affected values subsequently removed from the analysis files. Respiratory inductance plethysmography data signals of the abdominal and ribcage bands were filtered using a 201 sample median filter to reduce electrical noise, artifact, and other disturbances to the belt while maintaining the integrity of the respiration waveform. The frequency of waveforms was used to calculate respiratory rate (RR). The voltage of waveforms from both belts were combined to create a representation of tidal movement (Emeriaud et al., 2008). A 1‐min calibration period during baseline was used to determine the standard deviation (SD) of the data and calculate the necessary coefficients for the ribcage and abdominal bands to be combined into a single channel, using the equation: β/α = SD(Abdominal)/SD(Ribcage) (Emeriaud et al., 2008; Sackner et al., 1989). Relative TV index was found using the cyclic height of the calculated tidal waveform. As an additional check, breaths were rejected if at any time either the abdominal or ribcage waveform hit full scale limit (<1% or >99%). There was an export function within the PVT‐Research Tool application, providing mean reaction time in milliseconds for each test. All variables were averaged over the 15‐min period at each altitude level, except SpO_2_ nadir which was calculated as the minimum SpO_2_ recorded during the 15‐min period. We also performed sensitivity analyses to determine if results were different based on data from the first or second half of this period. Weather data was obtained for barometric pressure at ground level (Weather Underground, website: www.wunderground.com), and corrected for temperature and humidity using the Antoine formula. Barometric pressures and PAO_2_ were estimated for each altitude level using the barometric pressure equation and alveolar gas equation respectively. SpO_2_ predictions from the estimated PAO_2_ values were derived using the Hill equation based on the oxygen‐hemoglobin dissociation curve (Hill et al., 1910), which does not account for physiological compensation in humans.

### 2.4 Statistical analysis

A sample size calculation was not conducted due to the pilot nature of the study. The effect of altitude (within‐subjects) was assessed using a repeated measures ANOVA to compare the control condition at ground, 8,000 feet, and 12,500 feet. Multiple comparisons were assessed using post hoc analyses with Bonferroni correction. A paired *t*‐test was used to compare the within‐subjects effect of BPAP vs. control at 8,000 feet and 12,500 feet. Based on our directional hypothesis for SpO_2_, a one‐tailed test was performed. A two‐tailed test was employed for all other variables. Continuous variables are reported as mean ± SD. A p‐value <0.05 was considered statistically significant. Analyses were performed with SPSS Statistics (Version 28.0, IBM Corp, NY, USA).

## 3 RESULTS

### 3.1 Participants

Ten participants (age: 47 ± 14; 50% female) were recruited and enrolled in the study. Participant characteristics are outlined in Table 1. A transcranial doppler ultrasound signal was able to be obtained in nine of the 10 participants (obtained in four males and five females).

**TABLE 1:** Participant characteristics.

Characteristic	Males (*n* = 5)	Females (*n* = 5)
Age (years)	45 ± 14	49 ± 16
Recreational flight hours	2410 ± 2633	489 ± 856
History of smoking (n)	2	1
History of Covid‐19 (n)	3	2
Body mass index (kg/m^2^)	29.0 ± 2.7	25.7 ± 2.6
Body fat (%)	25.2 ± 2.5	38.6 ± 6.8
FVC (L)	5.16 ± 0.75	3.58 ± 0.43
FVC (% predicted)	97 ± 8	98 ± 8
FEV_1_ (L)	3.88 ± 0.92	2.69 ± 0.28
FEV_1_ (% predicted)	90 ± 13	92 ± 10

*Note*: Data are mean ± standard deviation or number (*n*).

Abbreviations: FEV_1_ Forced Expiratory Volume in 1 s; FVC, Forced Vital Capacity.

### 3.2 Effects of altitude

**FIGURE 2:**
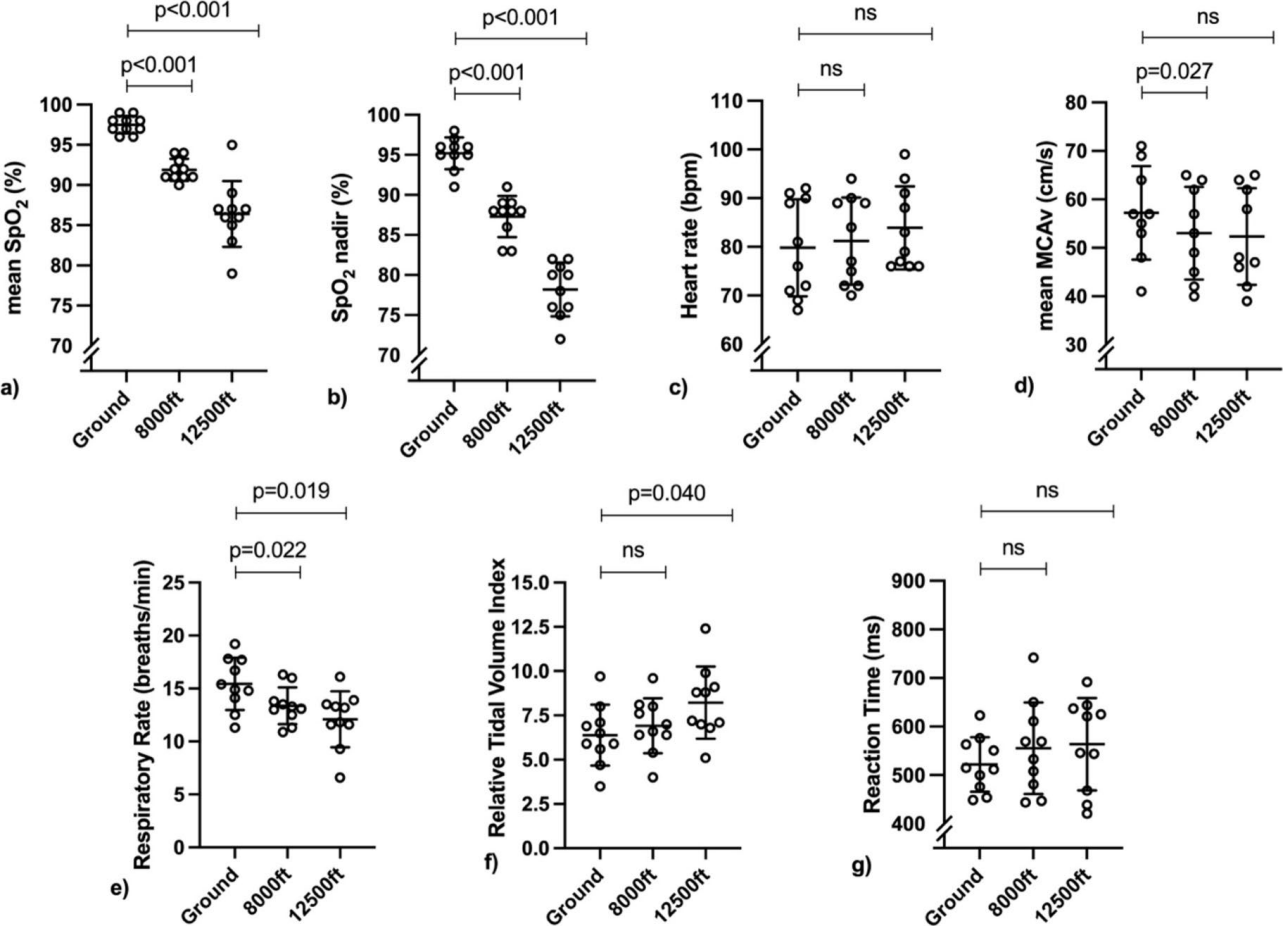
The effect of altitude at 8,000 feet (2438 m) and 12,500 feet (3810 m) on physiological responses compared with ground level. Data are analyzed by repeated measures ANOVA and presented as mean ± standard deviation. *p* values for post‐hoc comparisons are shown.

Table 2 outlines the barometric pressure at ground level and estimated barometric pressure, PAO_2_ and SpO_2_ at each altitude level. There was a significant effect of altitude on decreasing mean SpO_2_ (*p <* 0.001), SpO_2_ nadir (*p <* 0.001), mean MCAv (*p* = 0.019), and RR (*p =* 0.024), and increasing relative TV index (*p* = 0.005) and HR (*p* = 0.030). There was no effect of altitude on reaction time (*p* > 0.05, Figure 2). As shown in Figure 2, the significant reduction in mean SpO_2_ was −6 ± 2% [effect size (ES) = 3.4] at 8,000 feet (*p* < 0.001) and − 11 ± 4% [ES = 2.6] at 12,500 feet (*p* < 0.001). SpO_2_ nadir showed a significant reduction of −8 ± 3% [ES = 3.3] at 8,000 feet (*p* < 0.001) and − 17 ± 4% [ES = 2.3] at 12,500 feet (*p* < 0.001). The significant reduction in mean MCAv was −4 ± 4 cm/s [ES = 1.1] at 8,000 feet (*p* = 0.027) but not significant at 12,500 feet [−5 ± 6 cm/s; ES = 0.8]; [*p* = 0.149]. RR showed a significant reduction of −2 ± 2 breaths per minute at 8,000 feet [ES = 1.1] (*p* = 0.022) and − 3 ± 3 breaths per minute [ES = 0.9] at 12,500 feet (*p* = 0.019). Relative TV index showed a significant increase of 1.8 ± 1.9 [ES = 1.0] at 12,500 feet (*p* = 0.040) but no significant change at 8,000 feet [0.5 ± 1.0; ES = 0.5; *p* = 0.399]. The change in HR of 1 ± 2 bpm [ES = 0.7] at 8,000 feet and 4 ± 5 bpm [ES = 0.8] at 12,500 feet were not significant for post‐hoc comparisons (*p* = 0.199 and *p* = 0.094 respectively). Sensitivity analyses for the second half of each condition revealed no change in RR at 8,000 feet [MD = −1 ± 2 breaths per minute; *p* = 0.117] or 12,500 feet [MD = −3 ± 3 breaths per minute; *p* = 0.057]. Results for other variables were unchanged.

**TABLE 2:** Barometric pressures at ground level with estimations for barometric pressure, PAO_2_ and SpO_2_ at each altitude level.

Characteristic	Ground level	8,000 feet	12,500 feet
Barometric pressure (mmHg) Measured	736 ± 1	–	–
Barometric pressure (mmHg) Corrected for humidity and temperature	711 ± 6	–	–
Barometric pressure (mmHg) Estimated (from corrected)	–	566 ± 1	487 ± 2
PAO_2_ (mmHg) Estimated	95 ± 0.3	59 ± 0.5	42 ± 0.7
SpO_2_ (%) Estimated	95%	88%	75%
Mean SpO_2_ (%) Measured	98 ± 1.1	92 ± 1.4	86 ± 4.1
Nadir SpO_2_ (%) Measured	95 ± 2.0	87 ± 2.6	78 ± 3.4

*Note*: Data are mean ± standard deviation.

Abbreviations: PAO_2_, Alveolar partial pressure of oxygen; SpO_2_, Peripheral oxygen saturation.

### 3.3 Effects of BPAP versus control

**FIGURE 3:**
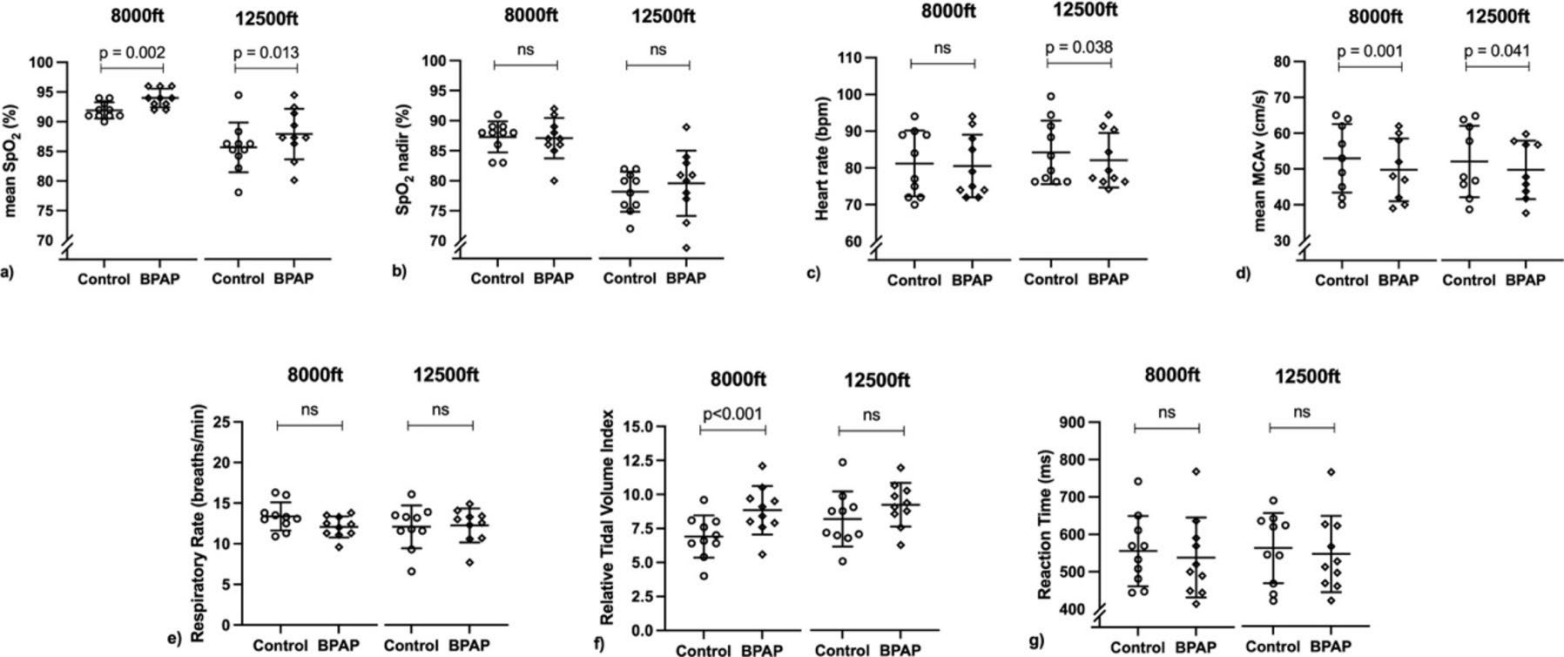
The effect of bi‐level positive airway pressure (BPAP) compared with control on physiological responses at 8,000 feet (2438 m) and 12,500 feet (3810 m). Data are analyzed by paired t‐tests and presented as mean ± standard deviation. Compared with control, BPAP significantly increased (a) mean peripheral oxygen saturation (SpO_2_) at 8,000 feet and 12,500 feet, (c) heart rate at 12,500 feet only, (d) mean middle cerebral artery velocity (MCA*v*) at 8,000 feet and 12,500 feet, and (f) relative tidal volume index at 8,000 feet only. There was no significant effect (ns) of BPAP compared with control (*p* > 0.05) on (b) nadir SpO2, (e) respiratory rate, or (g) reaction time.

There was a significant effect of BPAP compared with control on mean SpO_2_ at 8,000 feet (mean difference [MD] = 2 ± 2%; *p* = 0.002; ES = 1.3) and 12,500 feet (MD = 2 ± 3%; ES = 0.8; *p* = 0.013) (Figure 3). There was also a significant effect of BPAP on reducing MCAv at 8,000 feet (MD = −3 ± 2 cm/s; ES = 1.6; *p* = 0.001) and 12,500 feet (MD = −2 ± 3 cm/s; ES = 0.8; *p* = 0.04) (Figure 3). BPAP significantly increased relative TV index at 8,000 feet (MD = 1.9 ± 0.8; ES = 2.3; *p* < 0.001) but the increase was not significant at 12,500 feet (*p* > 0.05, Figure 3). HR was significantly lowered by BPAP at 12,500 feet (MD = ‐2 ± 3 bpm; ES = 0.8; *p* = 0.038) but not 8,000 feet (*p* > 0.05, Figure 3). There was no effect of BPAP (*p* > 0.05) on SpO_2_ nadir, RR, or reaction time (Figure 3). Sensitivity analyses for the second half of each condition revealed no effect of BPAP compared with control on mean SpO_2_ at 8,000 feet (MD = 1 ± 2%; ES = 0.5; *p* = 0.061) or 12,500 feet (MD = 1 ± 2%; ES = 0.4; *p* = 0.114). Results for other variables were unchanged.

## 4 DISCUSSION

The primary aim of this pilot randomized cross‐over study was to investigate whether positive pressure ventilation delivered via a commercially available BPAP device, could improve SpO_2_ without supplemental oxygen, during flight in an unpressurized aircraft at altitudes commonly experienced by general aviation pilots (8,000 feet and 12,500 feet). In line with our hypothesis, the main finding was that BPAP significantly increased mean SpO_2_ compared with control at 8,000 feet (2438 m) and 12,500 feet (3810 m).

While there has been extensive research into the effects of positive pressure breathing to protect pilots at extreme altitudes and improve tolerance of +Gz acceleration forces (Fong & Fan, 1997; Glaser et al., 2017), we report on a novel study to investigate the use of positive pressure ventilation during general recreational aviation. Early work in the 1940's used moderate levels of airway pressure (23 mmHg = 30 cmH_2_O) for their focus on altitude protection, compared with more recent work involving the study of higher airway pressures (up to 80 mmHg = 110 cmH_2_O) to tolerate the high Gz capabilities of modern aircraft (Glaser et al., 2017). This prior work has predominately used continuous positive pressure breathing in combination with increased fraction of inspired oxygen. In contrast, the current study used BPAP with low airway pressures (IPAP: 10 cmH_2_O and EPAP: 5 cmH_2_O) without increasing the fraction of inspired oxygen, and found a significant increase in mean SpO_2_ compared with control at both 8,000 feet (2438 m)and 12,500 feet (3810 m). Since gravity can exert a ventilation gradient, whereby there is heterogeneity in the distribution of ventilation favoring the lower parts of the lung (Galvin et al., 2007; West, 1969), the added pressure from BPAP may increase total lung volume allowing for better distribution of air, thus improving homogeneity of alveolar ventilation (British Thoracic Society, 2002). Indeed, we found a significant increase in the relative TV index with BPAP at 8,000 feet.

The improvement in mean SpO_2_ levels with BPAP compared with control, while significant, were numerically modest with mean improvements from 94% to 96% at 8,000 feet (2438 m) and 86% to 88% at 12,500 feet (3810 m). Furthermore, this modest effect was no longer significant during the second half of flight at 8,000 and 12,500 feet (after 7 min), and there was no effect of BPAP on nadir SpO_2_. The importance of nadir SpO_2_ compared with mean SpO_2_ is this study's context is unknown, however we believe mean SpO_2_ is more representative of the overall stimulus. Moreover, a drop in nadir SpO_2_ could reflect a very brief desaturation due to change of body position or peripheral vasoconstriction. Nonetheless it should be noted, that in an operational setting the SpO_2_ nadir may indicate the greatest degree of hypoxia and presence of risk, and we found no effect of BPAP on the nadir response. Given the pilot nature of this study we used low airway pressures (IPAP: 10 cmH_2_O and EPAP: 5 cmH_2_O) when compared with previous work (30–110 cmH_2_O) outlined above (Glaser et al., 2017). Our low airway settings are consistent with initial doses of positive pressure ventilation administered in a clinical setting (Alviar Carlos et al., 2018). Higher airway pressures (e.g., IPAP: 13–15 cmH_2_O and EPAP: 8–10 cmH_2_O) may provide greater improvements in SpO_2_ or for a longer duration. Commonly accepted levels for clinical ventilation are up to 30–35 cmH_2_O with home ventilation devices typically providing ~10–30 cmH_2_O (Kushida et al., 2008). Moreover, CPAP may provide greater improvements in SpO_2_ than BPAP during these general aviation altitudes, given CPAP is primarily used to improve oxygenation while BPAP is used to improve ventilation. We used BPAP as opposed to CPAP since BPAP exerts a lower pressure during expiration (compared with inspiration) which may prevent over‐distention of the of lungs. This lower device pressure during expiration can reduce the effort required by the participant during exhalation and hence reduce the burden, fatigue, and discomfort (Barach et al., 1946). Also, anecdotally during study planning, we found BPAP had a greater effect on SpO_2_ at altitude than CPAP.

We also found a small reduction in MCAv with BPAP compared with control. It should be noted that the reductions in MCAv with BPAP were small and the MD between BPAP and control was similar at 8,000 feet (2438 m) and 12,500 feet (3810 m). Early invasive work in anesthetized dogs (Beecher et al., 1943; Braunwald et al., 1957; Carr & Essex, 1946; Maloney Jr. & Whittenberger, 1957; Qvist et al., 1975) and human patients (Hobelmann Jr et al., 1975) at normal atmospheric pressure showed that positive pressure breathing caused elevations in central and jugular venous pressures, and reductions in arterial pressure, cardiac output, and central blood volume. Decreases in cardiac output and/or central blood volume with positive pressure ventilation could explain the reduced blood flow velocity through the MCA (Rickards et al., 2015). Beecher et al. (Beecher et al., 1943) found that positive pressure breathing decreased flow in the femoral and carotid arteries of dogs, and Kety and Schmidt (Kety & Schmidt, 1946) showed passive hyperventilation (using a positive pressure device) decreased cerebral blood flow by ~30% in young men. More recently, Yiallourou et al. (Yiallourou et al., 2013) found that CPAP (delivered at 15 cmH_2_O) reduced MCAv and internal carotid artery flow compared with control in awake humans, which corresponded with a small reduction in transcutaneous carbon dioxide. Some studies have also found compensatory increases in HR to offset reductions in stroke volume to maintain cardiac output (Qvist et al., 1975). In contrast, we found a modest but significant reduction in HR with BPAP at 12,500 feet (3810 m) and no change in HR at 8,000 feet (2438 m). More recent work in humans has found the use of G‐suits, to provide counter pressure, can promote venous return during positive pressure breathing and maintain arterial pressures and cardiac output (Goodman et al., 1992; Han et al., 2002). Furthermore, Palasiewicz et al. (Palasiewicz et al., 1997) studied the acute effects of CPAP and BPAP on pulmonary haemodynamics, and found CPAP increased mean pulmonary intravascular pressures (which can hamper venous return and pulmonary blood flow) but found no effect with BPAP. Therefore, another benefit of BPAP over CPAP may be providing positive pressure ventilation with less effects on pulmonary haemodynamics and venous return.

Acute hypoxia causes a host of compensatory physiological processes to preserve homeostasis and protect vital organs. For example, hypoxia has been shown to increase HR, cardiac output, ventilatory drive, and cerebral blood flow (Ainslie et al., 2016; Harding & Mills, 1983; Wilson et al., 2011), although the magnitude of change in cerebral blood flow may be modified by the co‐existing hypocapnia from increased minute ventilation (Harding & Mills, 1983). We did find a significant increase in HR with altitude. In contrast, we found a decrease in RR with altitude at 8,000 feet (2438 m) and 12,500 feet (3810 m). Although, there was no significant change in RR during the second half of the condition, indicating participants breathing may have stabilized after several minutes at each altitude. While the acute response to hypoxia is an augmentation of ventilatory activity (both frequency rate and TV), a phenomenon of hypoxic ventilatory decline (also termed hypoxic ventilatory depression) has been described when hypoxemia is sustained for 5–30 min (Honda, 1995; Pamenter & Powell, 2016). This could have occurred in the present study given our 8,000 feet condition was 15 min followed by 12,500 feet condition for a further 15 min. Although, while we found a reduction in RR with altitude we found a concurrent increase in relative TV index, which may indicate total ventilation did not change. However, as we did not measure TV or ventilation directly, these data should be interpreted cautiously, and future studies should aim to include a direct measure of ventilation to further investigate these findings. Other reasons we may not have seen an increase in RR with altitude are due to the low altitude levels (i.e., ventilatory responses typically seen >10,000 ft) and/or reduced susceptibility to altitude (as there can be natural variation in the population).

We also found a decrease in MCAv with altitude at 8,000 feet (2438 m) during the present study, which was unexpected, but may be explained by the following. Firstly, during rapid ascents, there might be a preference for increasing blood flow within the vertebral artery (for delivery to the cardiorespiratory centres within the brainstem) as opposed to the internal carotid artery (i.e., delivers to the MCA) (Ainslie & Subudhi, 2014; Subudhi et al., 2014). We did not measure the posterior cerebral artery using transcranial doppler ultrasound, which receives blood flow from the vertebral artery. Secondly, with acute increases in altitude, the decreases in PaO_2_ occur concurrently with decreases in PaCO_2_ (Shaw et al., 2021), which have opposing effects on cerebral blood flow (Ainslie & Duffin, 2009; Ainslie & Subudhi, 2014). Within‐ and between‐individual variability in cerebral blood flow changes have been reported during acute/acclimatization periods at altitude given the various compensatory reflex mechanisms with opposing actions including the (1) hypoxic ventilatory response, (2) hypercapnia ventilatory response, (3) hypoxic cerebral vasodilation, and (4) hypocapnia cerebral vasoconstriction (Ainslie & Subudhi, 2014). The cerebral vasculature is exquisitely sensitive to PaCO_2_ as a vital homeostatic function to maintain central pH (Ainslie & Duffin, 2009; Chesler, 2003), therefore even small decreases in PaCO_2_ up to 10,000 feet or 3048 m (i.e., from 40 mmHg to 35–40 mmHg) could affect cerebral blood flow (Ainslie & Subudhi, 2014). In contrast, the role of PaO_2_ on the cerebral vasculature is relatively minimal until a certain threshold is reached to promote cerebral vasodilation, which is reported to be ~50 mmHg PaO_2_ (Ainslie et al., 2016) or 90% SpO_2_ (Gupta et al., 1997). While we did not measure PaO_2_ levels, Shaw et al. (Shaw et al., 2021) depicts that PaO_2_ levels do not drop to 40–60 mmHg until 10,000–15,000 feet (3048–4572 m), and the mean SpO_2_ of our participants did not drop below 90% until 12,500 feet. Our 15‐min exposure to 12,500 feet may not have been long enough to reach this threshold of hypoxia and stimulate an increase in MCAv. Although, the reduction in MCAv was only significant at 8,000 feet, not 12,500 feet (3810 m). While we found a reduction in RR with altitude this was matched with an increase in relative TV index, indicating a change to the ventilatory response. Therefore, in our study, a decrease in PaCO_2_ with altitude may explain the decrease in MCAv at 8,000 feet.

We did not find any effect of altitude or BPAP on reaction time using a 3‐min psychomotor vigilance test. Previous work assessing cognitive deficits at similar altitudes to our study have shown conflicting results (Hewett et al., 2010; Kelman & Crow, 1969; Nesthus et al., 1997). Kelman and Crow (Kelman & Crow, 1969) found performance on a vigilance task was impaired at 8,000 feet (2438 m). Furthermore, Nesthus et al. (Nesthus et al., 1997) found pilots flying at altitudes of 10,000–12,000 feet (3048–3658 m) without supplemental oxygen had an increase in procedural errors compared with pilots who had oxygen, although these pilots had been flying at ≥10,000 feet (3048 m) for 2 hours. In contrast, Hewett et al. (Hewett et al., 2010) did not find any cognitive impairments at 8,000, 10,000, or 12,000 feet (2438 m, 3048 m, 3658 m respectively) using a reduced oxygen breathing device to simulate altitude exposure. Conflicting results within the existing literature may be due to different tests assessing different aspects of cognitive function, which may not all be impacted by altitude. The psychomotor vigilance test used in the current study has historically been administered as a 10‐min test and used to assess attention and alertness in sleep loss or fatigue‐related research (Dinges & Powell, 1985). Although a 3‐min version has been validated to be sensitive to deficits from sleep loss and alcohol in aviation research (Benderoth et al., 2021), it may not have been long enough to detect deficits from short exposure to altitude (i.e., 7 min at 12,500 feet). A longer or more challenging test (e.g., with multi‐tasking), greater exposure to altitude, and/or larger hypoxic stimulus may be needed to detect impairments in reaction time. The practice of cognitive tests prior to flight, has also been shown to improve reaction time performance at altitude (Denison et al., 1966), which may have influenced our results. It is also possible that when under more challenging conditions, participants may work harder to be more focused and attentive to a task.

Our study is not without limitations. This was a small pilot study which limits our power to detect differences and the generalisability of findings. A sample size of 10 participants (50% females) was chosen to provide preliminary data so that effect sizes could be generated to inform a sample size calculation for future adequately powered studies. We were unable to obtain sufficient blood pressure data due to device error with altitude, and future studies should confirm their device can withstand changes in altitude. We did not measure end‐tidal CO_2_, PaCO_2_ or cardiac output, SpO_2_ was measured rather than PaO_2_, and our measure of TV was indirect, which limits our ability to confirm the mechanisms involved. For future studies, the direct measurement of arterial blood gases, respiratory exchange ratio, and ventilation will be important to confirm the ventilatory response to prolonged hypoxemia at altitude and understand underlying mechanisms (e.g., can the reduction of MCAv be explained by vasoconstriction induced by low PaCO_2_). Another limitation of using SpO_2_ is that hyperventilating can increase SpO_2_ without the same magnitude of effect on PaO_2_. This is another reason that directly assessing minute ventilation and arterial O_2_/CO_2_ will be important for future studies. Furthermore, the direct measurement of total atmospheric gases (e.g., O_2_, CO_2_), the partial pressures of O_2_, and BPAP pressures are recommended for future studies to confirm the exposure. We used MCAv as a surrogate for cerebral blood flow, which does not consider changes in vessel diameter that contribute to changes in flow. We used additional cognitive tests during the study including the Trail‐making B and Stroop tests, however despite providing participants with time for familiarization there were considerable practice effects, and therefore have not been included within the study outcomes.

### 4.1 Conclusion

This study provides preliminary results that BPAP may improve mean oxygen saturation for recreational aviators up to 12,500 feet (3810 m) without supplemental oxygen. Future adequately powered studies are needed to further investigate potential utility of positive pressure ventilation to improve oxygen saturation and cognition in recreational aviation.

## AUTHOR CONTRIBUTIONS

All authors contributed to Conceptualization or Study Design. Jenna L. Taylor, Aidan K. Downs, Crystal L. Marshall, J. Hunter Downs III, Josh Donkor, Jessica I. Johnston, Douglas Rozendaal, and Peter L. Larsen contributed to data collection. Jenna L. Taylor, Aidan K. Downs, and J. Hunter Downs III contributed to data analysis. Jenna L. Taylor and Crystal L. Marshall drafted the manuscript, and all other authors provided critical review of the manuscript. All authors approved the final version of the manuscript.

## FUNDING INFORMATION

No funding was received for this work.

## CONFLICT OF INTEREST STATEMENT

The author Douglas Rozendaal is the owner of PlaneLease LLC and declares no commercial interest with this study. The remaining authors declare that the research was conducted in the absence of any commercial or financial relationships that could be construed as a potential conflict of interest.

## ETHICS STATEMENT

The study was approved by the Institutional Review Boards from Mayo Clinic and MercyOne North Iowa Medical Center. All participants provided written informed consent prior to study participation.

## Data Availability

The authors confirm that the data supporting the findings of this study are available within the article (see Results section, Table 1, Figure 2, and Figure 3).
